# Protective effects of commercial artichoke (*Cynara scolymus L*.) leaf powder against aflatoxin B1-induced reproductive toxicity in male rats

**DOI:** 10.1007/s12550-025-00603-3

**Published:** 2025-08-13

**Authors:** Asmaa A. Aziz, Heba Abdelmegeed, Mokhtar I. Yousef, Doaa A. Ghareeb, Abeer El Wakil

**Affiliations:** 1 Department of Environmental Studies, Institute of Graduate Studies and Research, Alexandria University, Alexandria, Egypt; 2 Department of Chemistry of Natural Compounds, National Research Centre, Giza, Egypt; 3 Bioscreening and Preclinical Trial Lab, Biochemistry Department, Faculty of Science, Alexandria University, Alexandria, Egypt; 4 Department of Biological and Geological Sciences, Faculty of Education, Alexandria University, Alexandria, 21526 Egypt

**Keywords:** Natural product, Prostate-specific hormone, Follicle-stimulating hormone, Luteinizing hormone, Antioxidant

## Abstract

Aflatoxin B_1_ (AFB_1_) is an unavoidable environmental pollutant frequently found in feed and foodstuffs and is considered the most toxic of all aflatoxins, known to impair testicular function. This study investigated the potential protective effect of artichoke leaves powder (ArLP) against the reproductive toxicity induced by AFB_1_ in male rats. In a 42-day experiment, rats received either sterile water, 4% DMSO, ArLP (100 mg/kg/body weight), AFB_1_ (72 µg/kg/body weight), or a combination of ArLP and AFB_1_ via oral gavage. AFB_1_ exposure led to increased oxidative stress, abnormal sperm parameters, hormonal disturbances, elevated prostate specific antigen (PSA) levels and histopathological damage in the testes. Co-administration of ArLP with AFB_1_ significantly mitigated these adverse effects, with most parameters approaching values observed in the control groups. These findings suggest that oral administration of ArLP exerts a protective effect against AFB_1_-induced reproductive toxicity in male rats and support its potential use in mitigating toxin-related reproductive damage.

**Supplementary information:**

The online version contains supplementary material available at 10.1007/s12550-025-00603-3.

## Introduction

The ubiquitous presence of pollutants in the environment has been an increasing global concern (Pleadin et al. 2019). Mycotoxins, which are secondary metabolites produced by fungi, are among the most hazardous contaminants and, in some cases, exhibit greater toxicity than certain pesticides (Tarhane et al. 2023; Skrzydlewski et al. 2022; Ülger et al. 2020). These substances may be found in a variety of very important agricultural and food products depending on their physico-chemical characterization. They may be synthesized on the field, during harvest as well as during storage (Martín et al. 2022). Among these contaminats, aflatoxins produced by *Aspergillus* spp. deserve more attention because they are widely distributed in nature and represent big threats to food safety and human health (Jaćević et al. 2023). Aflatoxin B_1_ (AFB_1_) is deemed the most toxic of these compounds and its metabolites have a variety of adverse biological activities, including toxicity, teratogenicity, mutagenicity, and carcinogenicity (Tang et al. 2025; Cao et al. 2022; Pickova et al. 2021). Unavoidable exposure to AFB_1_ is associated with an increased incidence of negative health consequences, including reproductive disorders and dysfunction (Wang et al. 2025; Fasihi-Ramandi et al. 2023). Early alterations of reproductive toxicity are manifested by inhibition of gonadal sex determination gene and steroidogenesis, and disturbed gametogenesis, leading later to dysfunctions in sperm production (Hassan et al. 2022; Marcu et al. 2023). Such disrupted structures and functions of reproductive organs are evidenced by decreased fertility. In humans, it has been reported that about 30% of infertility reasons is caused by male factor, with 2% of these exhibiting suboptimal sperm parameters (Barratt et al. 2017). Nowadays, naturally derived products that counteract the adverse effects of variable toxicities are in the forefront line, particularly that the nature has been the source of life-changing and saving medications for centuries. In the present study, an approach to minimize reproductive toxicity caused by AFB_1_ was investigated in which diets were supplemented with an entirely natural, plant-based product, artichocke leaves powder (ArLP). This therapeutic alternative, if effective, would be eco-friendly, cost-effective and with minimal side effects. 

Natural products have been widely employed as a rich source of compounds for drug discovery and development with high molecular diversity, novel biofunctionality, and minimal adverse effects (El-Ashram et al. 2021; Amr et al. 2022; Naeem et al. 2022; Bakr et al. 2023; Abbas et al. 2024; Ali et al. 2024; Omar et al. 2024; Alqurashy et al. 2025a, 2025b; Zaied et al. 2025, Matar et al. 2025). Among these, artichoke (*Cynara scolymus*) is one of the potent herbal remedies as extracts obtained either from the whole plant or from different parts of artichoke mainly leaves, fruits, and roots. ArLP contains a wide range of bioactive compounds, including polyphenols and phenolic acids (Fig. S1), which were previously identified by reversed-phase HPLC in the same experimental series using the same batch of animals, dosing regimens, and ArLP extract. This identification aimed to evaluate the protective effects of artichoke against AFB1-induced neurotoxicity and hepatotoxicity (Ibrahim et al. 2022; Nasef et al. 2022), thereby highlighting its nutritional and pharmaceutical value. Moreover, the potential healh benefits of these leaves include, but is not limited to, anti-inflammatory, antioxidant, antibacterial, anticarcinogenic, hepatoprotective, choleretic, diuretic, and hypocholesterolemic activities (Awad et al. 2020; Salekzamani et al. 2019). Nevertheless, studies relating to the significance of ArLP potential in preventing and/or reducing reproductive toxicity still need to be explored.

In the current study, we aim to evaluate the potential protective effects of ArLP against AFB1-induced reproductive toxicity in male rats, an area that remains underexplored. For this purpose, adult male rats were orally administered ArLP (100 mg/kg body weight) and/or AFB_1_ (72 µg/kg body weight) for 42 consecutive days. We assessed a range of parameters related to male reproductive health, including biochemical markers, semen characteristics, reproductive hormones, and total prostate-specific antigen (PSA). In addition, oxidative stress markers and antioxidant enzyme activities were evaluated in testicular tissue, and histopathological examination was conducted to investigate tissue-level alterations.

## Materials and methods

### Chemicals

*Aspergillus flavus*’s AFB_1_ powder (C_17_H_12_O_6_) with 99.7% purity and 312.27 molecular weight (CAS Number 1162–65-8) was obtained from Sigma-Aldrich, France. Commercial capsules of artichoke leaf extract (Super Artichoke, capsules) were purchased from Western Pharmaceutical Industries, Cairo, Egypt. Other chemicals, solvents, and reagents of analytical grade were used throughout the whole study.

### Animals

Adult twenty-five male albino rats (185 ± 8 g) were purchased from the Medical Research Institute (Center of Medical Technology), Alexandria University, Egypt. The rats were randomly divided into five groups (5 rats/group) and housed in clean cages. For a 1-week acclimatization period, and throughout the entire treatment duration, animals had free access to clean tap water and a standard chow diet (composed of 7% simple sugars, 3% fat, 50% polysaccharide, and 15% protein (w/w); energy content: 3.5 kcal/g) supplied by a local manufacturer (Tanta feed Company, Egypt). The animals were maintained under standard housing conditions: temperature of 25 ± 3 °C, relative humidity of 50–70%, and a 12-h light/dark cycle.

### Experimental design

The experimental animal study was conducted for 42 consecutive days, during which five animals per group were orally administered different treatments as follows: the control group received 1 mL/kg body weight (BW) of sterile water; the DMSO group received 1 mL/kg BW of 4% dimethyl sulfoxide (DMSO); the AFB_1_ group was administered AFB_1_ at a dose of 72 µg/kg BW, corresponding to 1/100 of the LD_50_ (7.2 mg/kg BW); the ArLP group received 100 mg/kg BW of ArLP dissolved in 4% DMSO; and the final group (ArLP + AFB_1_ group) received both AFB_1_ and ArLP. AFB_1_ and ArLP were administered separately by oral gavage, each prepared in its respective vehicle. To avoid any potential pre-administration interaction, the two compounds were given at different time points on the same day, and doses were adjusted every second day according to the animal’s current body weight to ensure accurate and consistent dosing and minimize variability due to changes in weight during the course of treatment. All animals were then euthanized under general anesthesia using isoflurane (2 mL/kg BW) by inhalation, followed by cardiac puncture for blood collection. Blood plasma was isolated following centrifugation at 3000 rpm for 20 min. After removal of the reproductive sex organs (testes and epididymis) from the adhering fat and connective tissues, they were weighted, and the measure was recorded. Right testis from each animal was minced and homogenized (10%, w/v), separately, in ice-cold sucrose buffer (0.25 M) in a Potter–Elvehjem type homogenizer (Thomas Scientific, USA). The homogenates were centrifuged at 8000 rpm for 20 min at 4 °C, then the supernatants were collected for the determination of tested parameters.

### Animal ethics

All experimental procedures were approved by the Research Ethics Committee (Approval number: 10–230410-1–01) and conducted according to animal care and use guidelines stipulated by Alexandria University, Egypt, and strictly followed all the procedures and techniques used to minimize any suffering during the experiments. This study is reported in accordance with ARRIVE guidelines and all experiments were performed in accordance with these relevant guidelines and regulations.

### Biochemical analyses

#### Testes free radicals and antioxidant enzymes assays

The levels of thiobarbituric acid reactive substances (TBARS, Ref. code MD25 29), and the activities of key antioxidant enzymes, superoxide dismutase (SOD, Ref. code SD25 21), glutathione S-transferase (GST, Ref. code GT25 19), and glutathione peroxidase (GPx, Ref. code GP25 24), were determined using commercially available kits from BioDiagnostic Company (Egypt) according to the manufacturer’s instructions. The standardized published methods of glutathione level (GSH; Jollow et al. 1974), xanthine oxidase (XO) activity (Litwack et al. 1953), and the level of nitric oxide (NO, Montgomery and Dymock 1961) were applied.

#### Reproductive hormones

Enzyme-linked immunosorbent assay (ELISA) kits were used for the quantitative determination of concentrations in rat plasma of testosterone (DRG International Co., USA), follicle-stimulating hormone (FSH; Biocode-Hycel Co., Rue E. Solvay 101, 4000 Liège, Belgium), and luteinizing hormone (LH; Elabscience Biotechnology Co., Ltd). The assays were done strictly according to the procedure given along with the kits.

#### Total prostate-specific antigen

Prostate-specific antigen (PSA) was determined by an ELISA kit (Prechek Bio, Inc., USA). The assay system utilizes a goat anti-PSA antibody directed against intact PSA for solid phase immobilization (on the microtiter wells). Total PSA was measured with an immunocolorimetric assay using a microtiter plate reader with the optical density at 450 nm according to the manufacturer’s recommendations.

#### Semen characteristics

Epididymis was prepared to evaluate sperm count, sperm motility and sperm morphology. A Computer Assisted Semen Analysis (CASA System; Germany) with Olympus microscope (Olympus, Tokyo, Japan) was used according to the method of Adamkovicova et al. (2016). A total of 200 spermatozoa from each rat were examined and individually scored normal or abnormal, according to the strict sperm morphology criteria by Wang et al. (2016).

### Histopathological examination

The testes were assessed histologically using hematoxylin and eosin (H&E) stained sections where the tissues were immediately fixed in 10% formalin after collection and processed by cutting pieces in tissue cassettes with an automated tissue processor. They were then embedded in paraffin and sectioned with microtome at 4–6 µm thickness. The sections were then stained with hematoxylin and counterstained with eosin for histological examination. Images were taken on an Olympus XC30 microscope (Germany) with a digital Olympus UC30 camera (Germany). Representative pictures from each treatment group were taken at 20 × and 40 × magnification.

### Statistical analysis

All biochemical measurements were performed on five biological replicates per group, with each sample analyzed once without technical replication. Distribution of the data and homogeneity of variance was checked for all the parameters before analysis. The least significant difference test (ANOVA, LSD post hoc test, *p* < 0.05) was performed separately for each parameter. Body and reproductive sex organs mass and all biochemical parameters were expressed as the mean ± SE in the figures. Significant difference was *p < 0.05, **p < 0.01, ***p < 0.001, ****p < 0.0001. Statistical analysis was performed using GraphPad Prism 9.

## Results

### ArLP mitigates AFB1-induced impairment in body weight gain

**Fig. 1:**
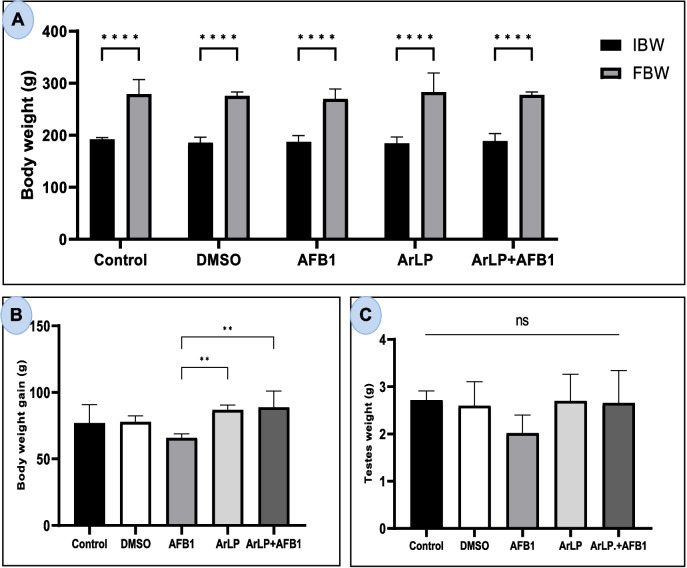
Body and testicular weights of control male rats and those treated with DMSO, ArLP, AFB1, or ArLP+AFB1 for 42 consecutive days. **A** Changes in body weight, showing initial body weight (IBW) is and final body weight (FBW), **B** body weight gain, and **C** testicular weight. Data are presented as mean ± SE (*n* = 5). Statistical analysis was performed using one-way ANOVA followed by LSD post hoc test. Significant differences are indicated as *p < 0.05, **p < 0.01, ***p < 0.001, ****p < 0.0001

We investigated the effects of ArLP on body and testicular weight as well as body weight gain. As demonstrated in Fig. 1A, each treatment group exhibited a significant increase between initial and final body weights over the course of the experiment, consistent with normal growth and food intake. However, no statistically significant differences were observed in either initial or final body weights when comparing across experimental groups. Regarding body weight gain, no significant differences were found among the control, DMSO, and ArLP groups, indicating that ArLP administration did not affect normal weight progression under the experimental conditions. However, there was a significant difference between AFB_1_ group and ArLP + AFB_1_ group (Fig. 1B). This significant increase in body weight gain of ArLP + AFB_1_ group compared with AFB_1_ group is attributed to ArLP administration. ArLP incorporation with AFB_1_ compensated for the observed decrease in body weight gain induced by AFB_1_ in AFB_1_-treated rats. Moreover, Fig. 1C shows that there was no significant difference between testicular weight among all groups. Hence, ArLP has no negative effect on body or sex organ weights. Furthermore, it reversed the decrease in body weight gain induced by AFB_1_ via improving food consumption. 

### ArLP normalizes oxidative stress markers and antioxidant defenses disrupted by AFB_1_

**Fig. 2:**
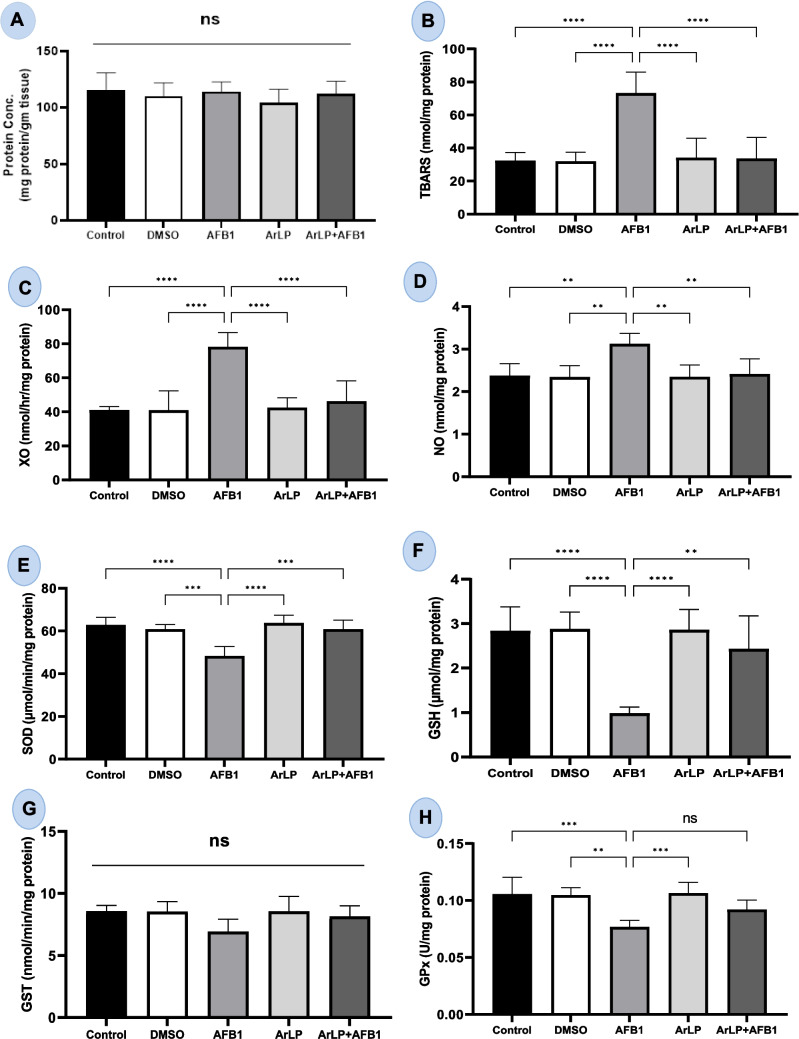
Assessment of antioxidant and oxidative stress markers in control male rats and those treated with DMSO, ArLP, AFB1, or ArLP+AFB1 for 42 consecutive days. **A** Protein content, **B** thiobarbituric acid reactive substances (TBARS), **C** xanthine oxidase (XO) activity, **D** nitric oxide (NO) concentration, **E** superoxide dismutase (SOD) activity, **F** reduced glutathione (GSH) concentration, **G** glutathione S-transferase (GST) activity, and **H** glutathione peroxidase (GPx) concentration.Data are presented as mean ± SE (*n* = 5). Statistical analysis was performed using one-way ANOVA followed by LSD post hoc test. Significant differences are indicated as *p < 0.05, **p < 0.01, ***p < 0.001, ****p < 0.0001

Total protein content in testicular tissue showed slight variations across experimental groups (Fig. 2A); however, these differences were not statistically significant. This indicates that under the present experimental conditions, neither AFB1 exposure nor ArLP treatment had a substantial effect on overall protein concentration, suggesting preserved tissue integrity at the general protein level.

We then examined the effects of ArLP, AFB_1_, and their combination on oxidative stress markers and antioxidant enzymes in testicular tissue. In the present study, the concentration of TBARS, a marker of lipid peroxidation, was used to assess oxidative stress in rat testes. TBARS levels were significantly increased in the AFB_1_-treated group compared to all other groups, while co-administration of ArLP with AFB_1_ significantly reduced TBARS concentrations (Fig. 2B). Similarly, XO activity (Fig. 2C) and NO concentration (Fig. 2D) were significantly elevated in the AFB_1_ group and significantly decreased in the ArLP + AFB_1_ group.

To assess antioxidant defense, the activity or concentration of key antioxidant markers, SOD, GSH, GST, and GPx, was evaluated in testicular tissue (Fig. 2E–H). ArLP administration alone did not significantly alter the levels of SOD, GSH, GST, or GPx compared with the control and DMSO groups. In contrast, AFB_1_ treatment led to a significant reduction in SOD activity, GSH, concentration, and GPx concentration.

Co-treatment with ArLP and AFB_1_ significantly increased SOD activity, GSH, and GPx concentrations compared to AFB_1_ group. GST levels showed a slight, but non-significant increase following ArLP or ArLP + AFB1 treatment.

### ArLP reverses AFB1-induced alterations in prostate-specific antigen and male sex hormone levels

To assess the effect of ArLP on male reproductive markers, PSA and sex hormone levels (LH, FSH, and testosterone) were measured.

**Fig. 3:**
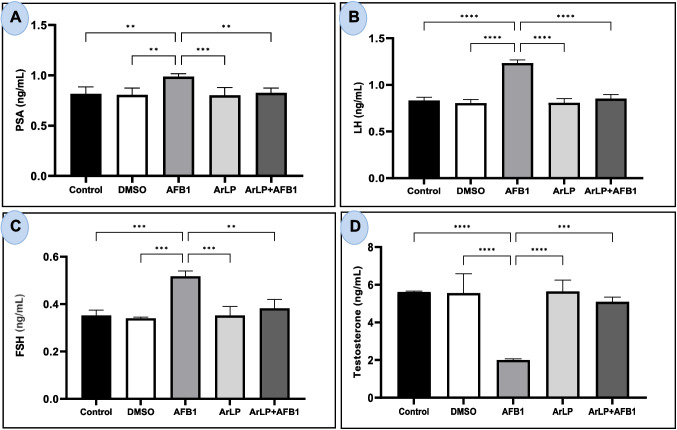
Analysis of male sex hormone and prostate specific antigen (PSA) levels in control rats and those treated with DMSO, ArLP, AFB1, or ArLP+AFB1 for 42 consecutive days. **A** PSA concentration, **B** luteinizing hormone (LH), **C** follicle-stimulating hormone (FSH), and **D** testosterone concentration. Data are presented as mean ± SE (*n* = 5). Statistical analysis was performed using one-way ANOVA followed by LSD post hoc test. Significant differences are indicated as *p < 0.05, **p < 0.01, ***p < 0.001, ****p < 0.0001

As shown in Fig. 3A, PSA concentrations did not differ significantly between the control, DMSO, and ArLP groups. However, PSA levels were significantly elevated in the AFB_1_-treated group compared to all other groups. Co-administration of ArLP with AFB_1_ significantly reduced PSA levels compared to AFB_1_ alone. Similar patterns were observed for LH and FSH. AFB_1_ treatment led to a significant increase in both LH (Fig. 3B) and FSH (Fig. 3C) levels, while ArLP + AFB1 treatment significantly reduced their concentrations compared to the AFB_1_ group.

Testosterone levels (Fig. 3D) remained unchanged in the control, DMSO, and ArLP groups. In contrast, AFB_1_ administration significantly decreased testosterone concentration compared to all other groups. Co-treatment with ArLP significantly restored testosterone levels compared to AFB_1_ treatment alone.

### ArLP partially ameliorates AFB1-induced impairments in semen quality

Semen characteristics, including sperm count, motility, and abnormal morphology, were assessed to evaluate the impact of ArLP, AFB1, and their combination on male reproductive function.

**Fig. 4:**
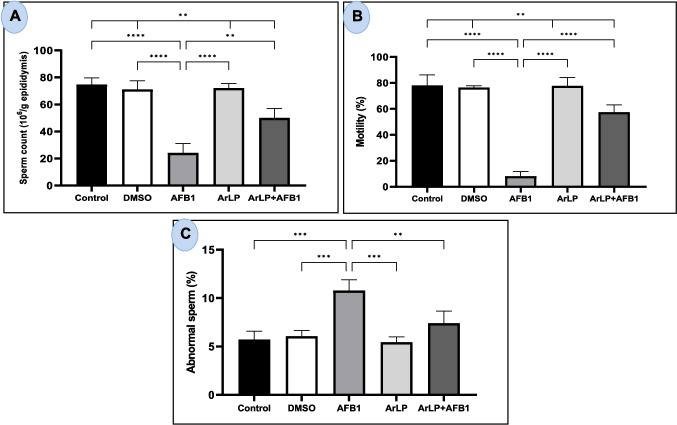
Semen analysis of control male rats and those treated with DMSO, ArLP, AFB1, or ArLP+AFB1 for 42 consecutive days. **A** Sperm count, **B** sperm motility (%), and **C** abnormal sperm (%). Data are presented as mean ± SE (*n* = 5). Statistical analysis was performed using one-way ANOVA followed by LSD post hoc test.Significant differences are indicated as *p < 0.05, **p < 0.01, ***p < 0.001, ****p < 0.0001

Sperm count in the ArLP group did not differ significantly from that of the control and DMSO groups (Fig. 4A). AFB_1_ treatment significantly reduced sperm count compared to all other groups. Co-administration of ArLP with AFB_1_ resulted in a significant increase in sperm count compared to the AFB_1_ group, though levels remained significantly lower than those of the control, DMSO, and ArLP groups.

As shown in Fig. 4B, AFB_1_ significantly reduced sperm motility compared to all other groups. The combination of ArLP and AFB_1_ significantly increased sperm motility relative to the AFB_1_-only group. However, motility levels in the ArLP + AFB_1_ group remained significantly lower than in the control, DMSO, and ArLP groups.

Abnormal sperm percentage was significantly elevated in the AFB_1_-treated group compared to all other groups (Fig. 4C). No significant difference in abnormal sperm percentage was observed among the control, DMSO, and ArLP groups. Co-treatment with ArLP significantly reduced the percentage of abnormal sperm compared to the AFB_1_ group, with values not significantly different from the control group.

### ArLP protects testicular tissue from AFB1-induced structural alterations

**Fig. 5:**
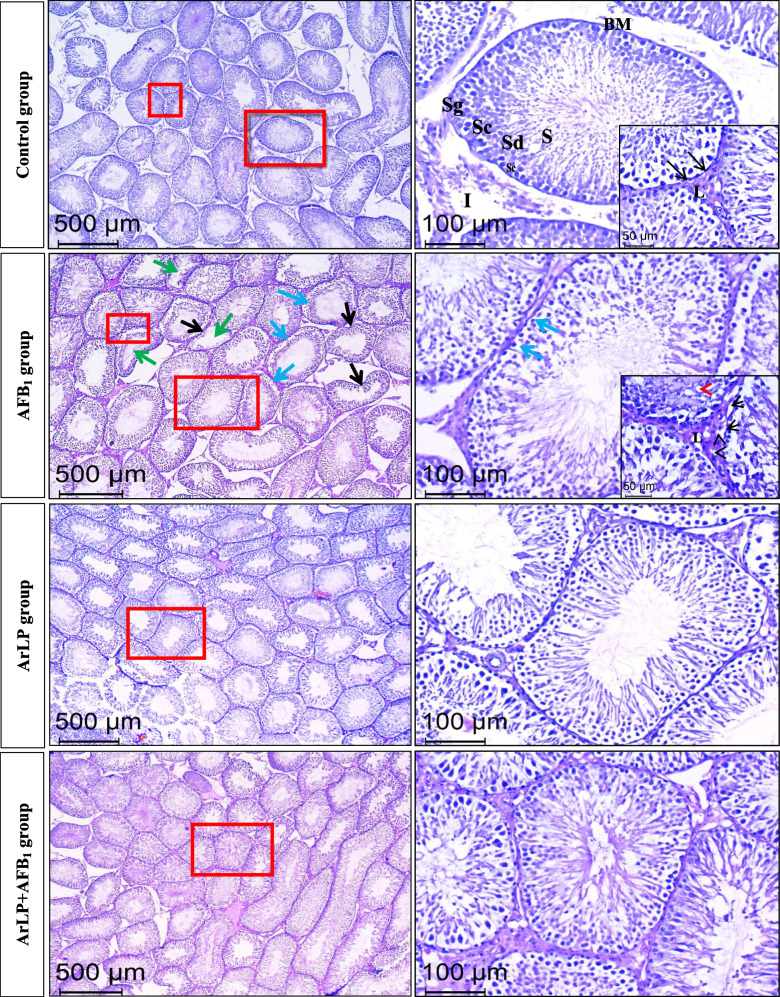
Representative images of H&E stained testicular tissue sections of rats from different experimental groups showing (i) in the control group, magnification of delineated squares revealed a regular basement membrane (BM, black arrows) with different stage of spermatogenesis: spermatogonia (Sg), primary and secondary spermatocytes (Sc); spermatids (Sd) and finally sperms (S). Leydig cells (L) are well seen in the interstitial space (I), (ii) in the AFB_1_ group, partially deformed or atrophied tubules (green arrows), irregular and buckled basement membrane (BM, black arrows), and reduced numbers of spermatogenic cells (blue arrows) were obvious. Magnification of delineated squares revealed vacuoles within both the tubules (red arrowhead) and the interstitial space (black arrowheads) among Leydig cells (L), (iii) in the ArLP group, magnification of delineated square shows no obvious histological damage on the seminiferous tubules as well as the interstitial spaces that display normal Leydig cells, and (iv) in the ArLP+AFB_1_ group, magnification of delineated square clarifies the impact of ArLP co-supplementation in alleviating the harmful effect of AFB_1_ on the seminiferous tubules manifested by the increase in the density of the different spermatogenic cells

ArLP remarkably improved semen characteristics; subsequently its effect on testicular tissue structure was examined. As illustrated in Fig. 5, the control group had normal architecture of seminiferous tubules showing normal basement membrane with different stages of spermatogenesis. This involved normal spermatogonia, primary and secondary spermatocytes, spermatids, and finally sperms. Besides, Leydig cells were well seen in the interstitial space. In ArLP group, no obvious histological damage was observed in both the seminiferous tubules and Leydig cells where they appeared normal as the control group. On the contrary, AFB_1_ administration affected the structure of the seminiferous tubules which became partially deformed or atrophied. The images revealed irregular and buckled basement membrane with mild testicular degeneration manifested by reduced numbers of spermatogenic cells. Other alterations observed were the presence of vacuoles within the tubules as well as in the interstitial space among Leydig cells. Moreover, increased luminal diameter which is one of the pathological features was clearly observed in AFB_1_ group. Combining ArLP with AFB_1_ counteracted the harmful effect of AFB_1_ administration on testicular tissue. This was evident by increasing the density of spermatogenic cells, decreasing the testicular lumen, and increasing the sperm filled lumens and the presence of normal Leydig cells compared with AFB_1_ group. Altogether, artichoke prevented testicular tissue deformities induced by AFB_1_ toxicity.

## Discussion

 Artichoke is well known for its nutritional benefits and medicinal properties. It exhibits various therapeutic advantages including hypoglycemic (Jalili et al. 2020), hepatoprotective (Nasef et al. 2022), anti-atherosclerotic and cholesterol lowering effects (Romain et al. 2018). Artichoke medicinal products are mainly composed of dried leaves. Therefore, we investigated the effects of ArLP against induced toxicity on male reproductive system by AFB_1_ which is known for imposing reproductive toxic consequences (Owumi et al. 2022).

 The beneficial components of ArLP, including polyphenols, phenolic acids, flavonols, flavonones, bioflavonids, and phenolic aldehydes, play an important role in health promotion as manifested by body weight gain. As expected, AFB_1_ caused a decrease in rats body weight, since weight loss is an indicator of a drop in animal’s health status. These findings are consistent with the data shown by other studies that used AFB_1_ orally (Subramaniam et al. 2022) or intraperitoneally (Deng et al. 2020; Ruggeberg et al. 2020). Upon ArLP administration, there was a significant increase in body weight gain of ArLP+AFB_1_ group compared with AFB_1_ group which clearly shows the positive effect of ArLP incorporation. The same effect of ArLP was reported in CCl4-intoxicated rats where ArLP significantly increased the body weight gain (Al-Ahdab 2014). In addition, no significant difference in testicular weight was observed among all groups.

 Furthermore, we investigated the effect of ArLP administration on antioxidant enzymes of testicular tissue due to their eminent role in protecting male reproductive system and testicular function (Barati et al. 2020; Mannucci et al. 2022). It was shown that AFB_1_ can induce oxidative stress and significantly impair the testicular antioxidant defense system causing germ cell apoptosis and spermatogenesis obstruction in both rats and mice (Yasin et al. 2018). The negative impact of AFB_1_ on the antioxidant system was evident even across species (Ben Taheur et al. 2022; Gao et al. 2022; Lin et al. 2022). One of the mechanisms responsible for AFB_1_ destructive action is oxidative stress related PI3K/AKT/mTOR signaling pathway in testicular tissue (Huang et al. 2019). In our study, AFB_1_ caused a significant decrease in SOD, GSH, GST, and GPx levels and combining ArLP with AFB1 led to significant elevation in their concentration in the testicular tissue. Similar results were reported by others in rats (Yilmaz et al. 2017) and in mice (Choudhary and Verma 2005) intoxicated with AFB_1_. On the other hand, ArLP showed the same improvement in antioxidant enzymes levels in other tissues such as brain (Ibrahim et al. 2022) and liver (Nasef et al. 2022). Moreover, this robust elevation in antioxidants activity induced by artichoke was also reported in vitro (Miláčková et al. 2017) and even in humans (Panahi et al. 2018). A meta‐analysis study provided convincing evidence for the increase in artichoke induced antioxidant activity in animals (Salekzamani et al. 2019). Our study also revealed that artichoke protected rats’ testicular tissue from the increase in TBARS and NO levels in addition to XO activity which was induced by AFB_1_. These data are consistent with the findings from Baldissera et al. (2018) and Owumi et al. (2023) who demonstrated that AFB_1_ induced oxidative toxicity in rats via inducing NO level and XO activity.

 Another aspect that we investigated is artichoke’s effect on PSA and male sex hormones. Prostate gland performs vital functions for supporting male fertility. Prostatic diseases or an unhealthy prostate can negatively affect normal male reproductive function (Verze et al. 2016). PSA is a well-known marker used for early detection of prostate cancer (Höti et al. 2023). Its high level is also detected in infertile men (Boeri et al. 2021). In our study, AFB_1_ induced a significant increase in PSA levels and ArLP administration along with AFB_1_ significantly decreased this elevated PSA to normal level. LH, FSH, and testosterone were also measured due to their immense importance for normal male reproductive function. LH induces testosterone release by the Leydig cells of the testes, and it initiates and maintains human spermatogenesis during puberty (Oduwole et al. 2021). On the other hand, FSH regulates the proliferation and maturation of germ cells (Oduwole et al. 2021). Testosterone is another vital hormone for the health status of male reproductive system. It is responsible for male morphological changes such as sex differentiation in addition to male physiological processes such as spermatogenesis and fertility (Grande et al. 2022). Herein, AFB_1_ led to a significant increase in LH and FSH levels in addition to a decrease in testosterone level when administered alone. However, ArLP administration along with AFB_1_ reversed AFB_1_-induced disturbance in the three male sex hormones. This was evident by the decrease in LH and FSH elevated levels as well as the increase in testosterone suppressed level and their return to normal levels. High LH and FSH levels are associated with low level of testosterone and testicular damage, respectively. The same pattern of hormonal changes was evident even in Wistar rat’s offspring which were exposed to AFB_1_ prenatally (Rotimi et al. 2021). This was also shown in humans who were exposed to AFB_1_ such as miller flour workers (Beshir et al. 2020). Although Owumi et al. (2022) group reported that there was a decrease in testosterone level as our study, they showed that LH has decreased after AFB_1_ administration. This may be attributed to the difference in AFB1 dose administered and the duration of exposure.

 Semen quality is another crucial determinant of male fertility. Hence, we examined the semen quality after AFB_1_ intoxication including sperm count, motility, and abnormality. AFB_1_ significantly decreased sperm count, almost diminished sperm motility and doubled the level of abnormal sperm. Our results indicate that while ArLP alone had no impact on sperm quality, its co-administration with AFB_1_ effectively attenuated AFB_1_-induced impairments in semen parameters. Data from other studies supported our findings where AFB_1_ negatively affected sperm viability (Komsky-Elbaz et al. 2018). Furthermore, the changes seen in our study such as decreased sperm count and deteriorated quality of sperm have been reported in AFB_1_-treated mice and rats (Asadpour et al. 2020; Supriya et al. 2014). Faisal et al. (2008) demonstrated that AFB_1_ induced sperm mitochondrial pathology which led to sperm abnormality. It also caused significant changes in sperm proteomic profile and decreased its fertilization competence. As for artichoke, the study conducted by Mohammed et al.’s (2020) group was consistent with our findings. They showed that it successfully alleviated the reduction in sperm quality induced by nandrolone decanoate.

 Furthermore, the alterations in testicular tissue induced by AFB_1_ administration were investigated as well. AFB_1_ administration led to partially deformed or atrophied seminiferous tubules, reduced numbers of spermatogenic cells, and increased the vacuoles within the tubules as well as increased luminal diameter. Combining ArLP with AFB_1_ counteracted the harmful effects of AFB_1_ administration and restored rats normal testicular tissue. Similar deleterious changes on testicular tissue were reported by others in AFB_1_ intoxicated rats, including but not limited to, reduced differentiation and spermiogenesis ratios, germ cells dissociation, deformed seminiferous tubules with edematous connective tissue and tubular depletion (Yasin et al. 2018; Asadpour et al. 2020). In addition, studies displaying similar protective effect of artichoke on testicular tissue were reported (Mohammed et al. 2020). Therefore, our study suggests that ArLP exerts a protective effect against AFB_1_-induced toxicity in the male reproductive system when administered concurrently.

## Conclusion

AFB_1_ is an unavoidable environmental pollutant frequently found in feed and foodstuffs. It is the most toxic among all aflatoxins and has been shown to severely impair testicular development and function. Collectively, our data demonstrate that AFB_1_ adversely affects multiple parameters of male reproductive health, including oxidative stress markers, antioxidant enzyme activity, PSA levels, sex hormone concentrations, semen quality, and testicular histoarchitecture. Co-administration of ArLP effectively attenuated these AFB_1_-induced alterations and helped restore the measured parameters toward normal values. These findings support the potential role of ArLP as a protective agent against AFB_1_-induced reproductive toxicity and highlight the need for further mechanistic and dose-optimization studies in preclinical models. 

## Supplementary information

Below is the link to the electronic supplementary material.

ESM 1 (DOCX 35.7 KB)

## Data Availability

All data generated and analyzed in this study are included in this article.
