# Supplementary material for: Protective effects of commercial artichoke (Cynara scolymus L.) leaf powder against aflatoxin B1-induced reproductive toxicity in male rats
Source: Mycotoxin Res. 2025 Aug 13;41(4):597–609. doi: 10.1007/s12550-025-00603-3 (PMC12611995; doi:10.1007/s12550-025-00603-3)
Supplement: Supplementary file 1 — (DOCX 35.7 KB) [file 12550_2025_603_MOESM1_ESM.docx]

**Fig. S1:** Reversed-phase HPLC analysis of artichoke leaves powder showing its secondary metabolites composition as follows **(A)** All classes, **(B)** Miscellaneous classes, **(C)** Polyphenols, **(D)** Phenolic acids, and **(E)** Phenols. This analysis was previously conducted and published as part of our earlier investigations into the neuroprotective and hepatoprotective effects of ArLP in rats exposed to AFB1 (Ibrahim et al., 2022; Nasef et al., 2022) and is included here as supplementary background to support the current study.
